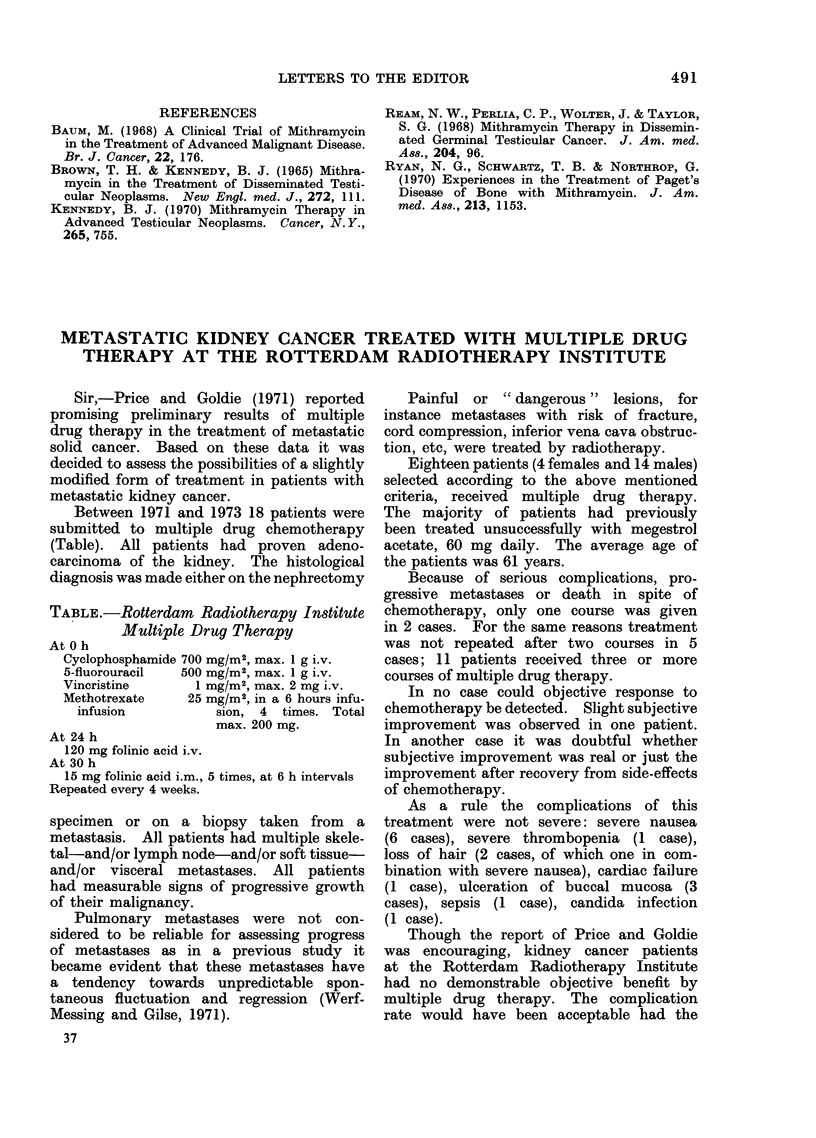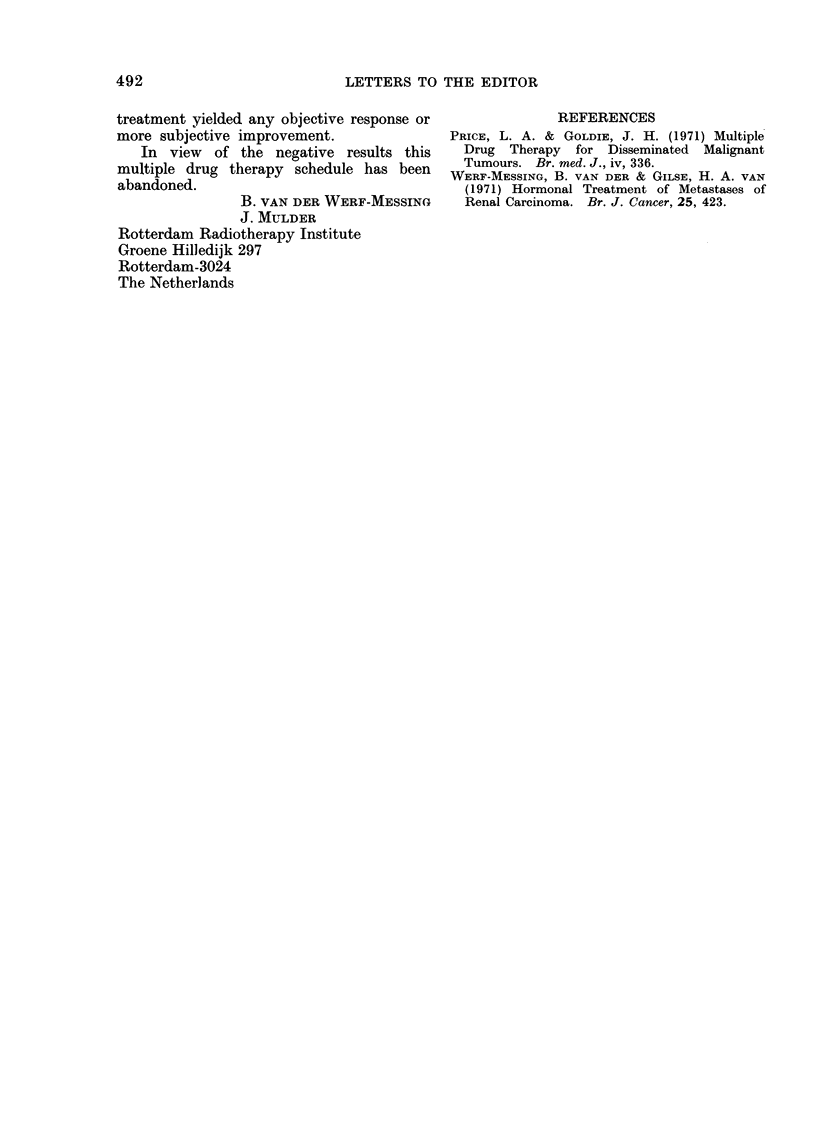# Letter: Metastatic kidney cancer treated with multiple drug therapy at the Rotterdam Radiotherapy Institute.

**DOI:** 10.1038/bjc.1974.104

**Published:** 1974-06

**Authors:** B. van der Werf-Messing, J. Mulder


### METASTATIC KIDNEY CANCER TREATED WITH MULTIPLE DRUG THERAPY AT THE ROTTERDAM RADIOTHERAPY INSTITUTE

Sir,—Price and Goldie (1971) reported promising preliminary results of multiple drug therapy in the treatment of metastatic solid cancer. Based on these data it was decided to assess the possibilities of a slightly modified form of treatment in patients with metastatic kidney cancer.

Between 1971 and 1973 18 patients were submitted to multiple drug chemotherapy (Table). All patients had proven adenocarcinoma of the kidney. The histological diagnosis was made either on the nephrectomy specimen or on a biopsy taken from a metastasis. All patients had multiple skeletal—and/or lymph node—and/or soft tissue—and/or visceral metastases. All patients had measurable signs of progressive growth of their malignancy.

TABLE.—*Rotterdam Radiotherapy Institute Multiple Drug Therapy*

| | |
|---|---|
| At 0 h | |
| Cyclophosphamide | 700 mg/m², max. 1 g i.v. |
| 5-fluorouracil | 500 mg/m², max. 1 g i.v. |
| Vincristine | 1 mg/m², max. 2 mg i.v. |
| Methotrexate infusion | 25 mg/m², in a 6 hours infusion, 4 times. Total max. 200 mg. |
| At 24 h | |
| 120 mg folinic acid i.v. | |
| At 30 h | |
| 15 mg folinic acid i.m., 5 times, at 6 h intervals | |
| Repeated every 4 weeks. | |

Pulmonary metastases were not considered to be reliable for assessing progress of metastases as in a previous study it became evident that these metastases have a tendency towards unpredictable spontaneous fluctuation and regression (Werf-Messing and Gilse, 1971).

Painful or "dangerous" lesions, for instance metastases with risk of fracture, cord compression, inferior vena cava obstruction, etc, were treated by radiotherapy.

Eighteen patients (4 females and 14 males) selected according to the above mentioned criteria, received multiple drug therapy. The majority of patients had previously been treated unsuccessfully with megestrol acetate, 60 mg daily. The average age of the patients was 61 years.

Because of serious complications, progressive metastases or death in spite of chemotherapy, only one course was given in 2 cases. For the same reasons treatment was not repeated after two courses in 5 cases; 11 patients received three or more courses of multiple drug therapy.

In no case could objective response to chemotherapy be detected. Slight subjective improvement was observed in one patient. In another case it was doubtful whether subjective improvement was real or just the improvement after recovery from side-effects of chemotherapy.

As a rule the complications of this treatment were not severe: severe nausea (6 cases), severe thrombopenia (1 case), loss of hair (2 cases, of which one in combination with severe nausea), cardiac failure (1 case), ulceration of buccal mucosa (3 cases), sepsis (1 case), candida infection (1 case).

Though the report of Price and Goldie was encouraging, kidney cancer patients at the Rotterdam Radiotherapy Institute had no demonstrable objective benefit by multiple drug therapy. The complication rate would have been acceptable had the treatment yielded any objective response or more subjective improvement.

In view of the negative results this multiple drug therapy schedule has been abandoned.

B. VAN DER WERF-MESSING
J. MULDER
Rotterdam Radiotherapy Institute
Groene Hilledijk 297
Rotterdam-3024
The Netherlands

REFERENCES

PRICE, L. A. & GOLDIE, J. H. (1971) Multiple Drug Therapy for Disseminated Malignant Tumours. *Br. med. J.*, iv, 336.

WERF-MESSING, B. VAN DER & GILSE, H. A. VAN (1971) Hormonal Treatment of Metastases of Renal Carcinoma. *Br. J. Cancer*, **25**, 423.